# Airbag-Related Ocular and Facial Trauma in Unrestrained Pediatric Front-Seat Passengers: A Report of Two Cases

**DOI:** 10.7759/cureus.110581

**Published:** 2026-06-10

**Authors:** Rattan Singh, Jyoti Barwa, Sahil Thakral, Ajay Kumar

**Affiliations:** 1 Forensic Medicine and Toxicology, All India Institute of Medical Sciences, Bathinda, Bathinda, IND; 2 Forensic Medicine and Toxicology, Dr S. S. Tantia Medical College Hospital and Research Centre, Rajasthan, IND

**Keywords:** airbag, automobile, burn injuries, facial, safety protocols

## Abstract

Airbags are considered a crucial safety feature in vehicles, offering significant protection by cushioning the occupants during collisions. During a collision, sudden deceleration or impact within a specified angle relative to the vehicle's longitudinal axis triggers crash sensors throughout the vehicle. These sensors send an electrical signal to the airbag cartridge, which rapidly inflates the nylon-rubber airbag within milliseconds, followed by a rapid deflation. However, they pose a risk of injury during their deployment if standard vehicle safety recommendations are not followed. Both external and internal injuries associated with airbag deployment have been reported, affecting drivers and passengers alike during crashes and sometimes leading to severe harm. This case involves two siblings, a six-year-old boy and a nine-year-old girl, who sustained ocular and facial injuries during an airbag deployment event while being seated on the front passenger side of a four-wheeled vehicle. Strict adherence to safety guidelines is essential when traveling in any vehicle to minimize the risk of severe or life-threatening injuries. Airbag design and technological enhancements are crucial to improving safety and reducing injuries from airbag deployments.

## Introduction

Airbags are life-saving devices that protect vehicle occupants during collisions, earning global recognition as an essential safety feature in modern automobiles. These devices consist of a coated nylon bag, typically housed in the steering wheel for the driver, on the dashboard for front-seat passengers, and within the doors for rear-seat occupants [1]. Vehicles can be equipped with both front and side airbags. While frontal airbags have been standard in passenger vehicles for over a decade, the number of mandatory side airbags varies across countries, depending on local legislative regulations [2]. Airbags are crucial for protecting occupants from hard interior surfaces, especially shielding the driver from the steering wheel, windshield, or dashboard during a collision [3]. While airbags are designed as safety devices to reduce the risk of death and serious injury, they can also pose a potential risk of harm upon deployment. This risk is particularly evident when safety guidelines are not followed, such as when airbags are deployed without seat belts, when children occupy the front seat, or when airbags are incompatible with child safety seats [4].

The performance of airbag systems depends on several factors, including material quality, deployment mechanisms, and testing protocols [5]. While airbag deployment plays a vital role in vehicle safety, it can also cause various injuries such as burns, ocular trauma, and blunt cerebrovascular damage during motor vehicle collisions (MVCs) [6]. Such airbag deployment anomalies can primarily result from impact-related sensor miscalculations (pseudo-collisions) or purely electronic/mechanical malfunctions unrelated to collisions. In the first scenario, crash sensors (accelerometers or pressure sensors) register a localized, rapid spike in deceleration or pressure change, usually triggered by a jarring event such as hitting a deep pothole. Hence, the software algorithm fails to differentiate this specific waveform from a structural crash, crossing the preprogrammed deployment threshold [7,8]. In the second scenario, the vehicle's physical environment remains stable. Still, an internal component failure in the form of an electrical short circuit, a corrupted software loop within the airbag control unit (ACU), or a corrupted signal can bypass the sensor logic entirely, thereby directly delivering the electrical current required to ignite the pyrotechnic inflator propellant [9,10]. Triggering of this mechanism can occur due to internal hardware deterioration, such as a failing sensing and diagnostic module; moisture corrosion shorting out wiring harnesses; or sudden braking deceleration forces that a properly functioning system should easily recognize as normal driving behavior [8,9].

This report describes a case of two siblings who suffered ocular and facial injuries during an airbag deployment event while seated in the front passenger seat of a four-wheeled vehicle. Although airbags greatly reduce fatalities and severe injuries in MVCs, they can also pose significant risks in the event of malfunction or an unexpected deployment, underscoring the need for ongoing research and enhanced medical protocols to manage deployment-related injuries. Sparse literature exists regarding pediatric trauma resulting strictly from apparent non-collision system malfunctions [11-13]. We highlight how this case contributes novel insights to the existing medical literature.

## Case presentation

This report describes a case involving two siblings who suffered airbag-related injuries while seated in the front passenger seat of a four-wheeled vehicle. A few moments before the incident, the younger sibling moved from the rear seat to sit beside her elder sister on the front passenger seat to her right; neither child was wearing a seatbelt. They were brought to a tertiary care hospital within three hours of the incident. Their father, who was driving at about 10 to 20 km/h, suddenly applied the brakes to avoid an on-road obstruction, causing the vehicle to stop abruptly and triggering an unexpected deployment of the frontal airbags on both sides. While the driver’s airbag remained intact and deflated after a few moments, the passenger-side airbag burst, causing injuries to the children. The children’s father, who was sitting on the driver's seat, and the mother, who was sitting on the rear passenger seat, remained unharmed. The four-wheeled vehicle was a five-seater compact sport utility vehicle manufactured in 2020. Although the vehicle was equipped with six standard airbags (dual front, dual front seat-mounted, and full-length side curtain), upon inquiry, it was revealed that none of the remaining four airbags deployed despite the sudden halt.

Case 1

**Figure 1:**
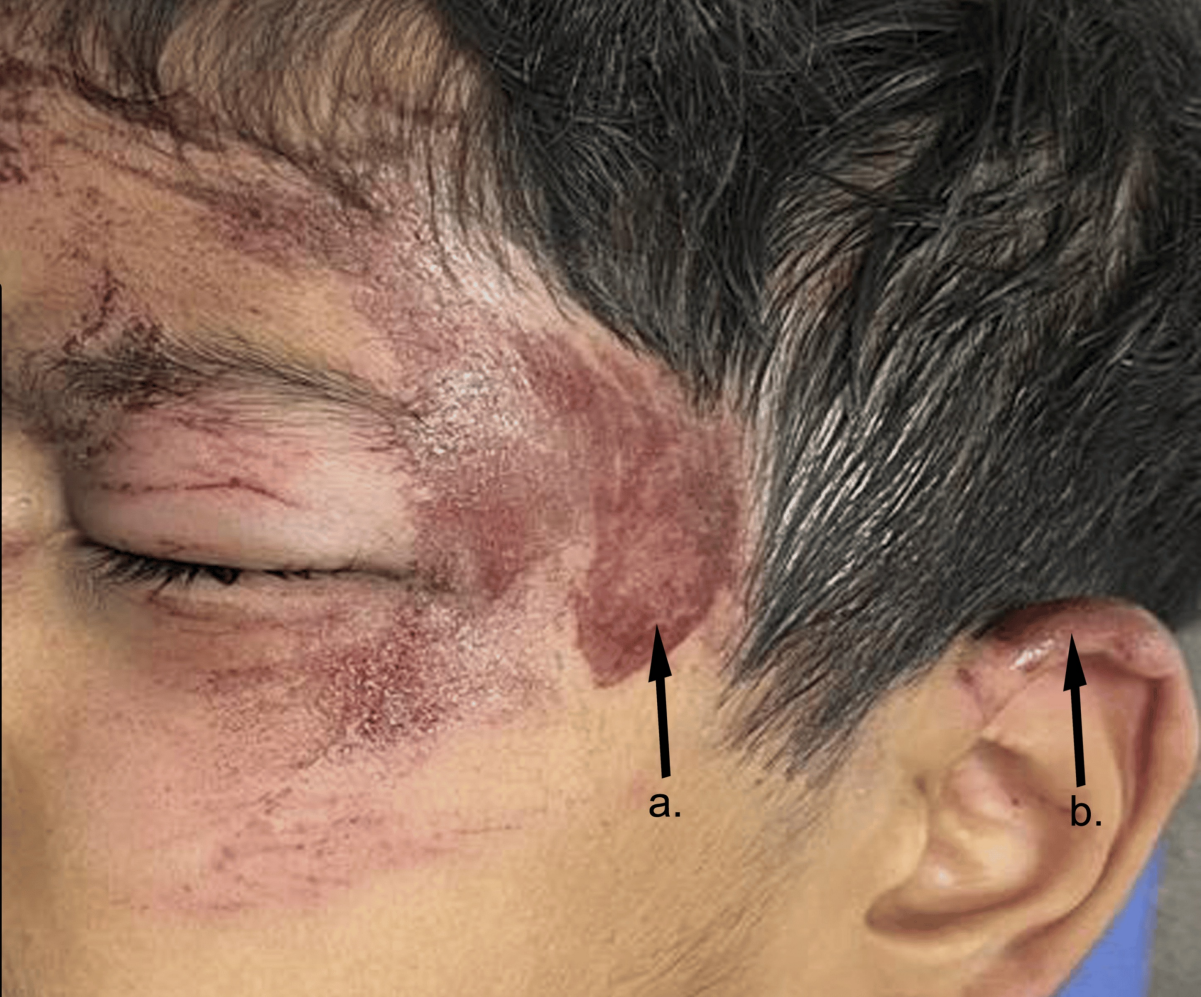
Facial injuries in case 1 (a) Superficial burn with redness and signs of inflammation. (b) Multiple abrasions. Informed consent was obtained from the patient’s legal guardian for the publication of the patient’s clinical information and images in this open-access journal.

A six-year-old boy arrived with stable vital signs but was uncooperative. He was breathing spontaneously with 100% oxygen saturation and had a Glasgow Coma Scale (GCS) score of 15. The Extended Focused Assessment With Sonography in Trauma (E-FAST) scan showed no abnormalities. On external examination, first-degree burn marks with multiple reddish scabbed abrasions were present over an area of 15.2 cm x 8.2 cm on the upper half of the left side of the face, situated below the hairline; the injury extended to the left side, involving the upper half of the pinna of the left ear. There was redness and signs of inflammation; no blisters were observed (Figure 1). All systemic examinations were within normal limits. X-rays of the chest, abdomen, and pelvis showed no abnormalities or skeletal injuries. There was periorbital edema in both eyelids; the left eye showed conjunctival congestion and chemosis. Comprehensive ophthalmic examination revealed normal visual acuity and movements in both eyes; fundal glow was present on ophthalmoscopy. A provisional diagnosis of left limbal ischemia with corneal opacity was made, and the patient was treated with oral antipyretics, topical antibiotics, and steroids.

Case 2

**Figure 2:**
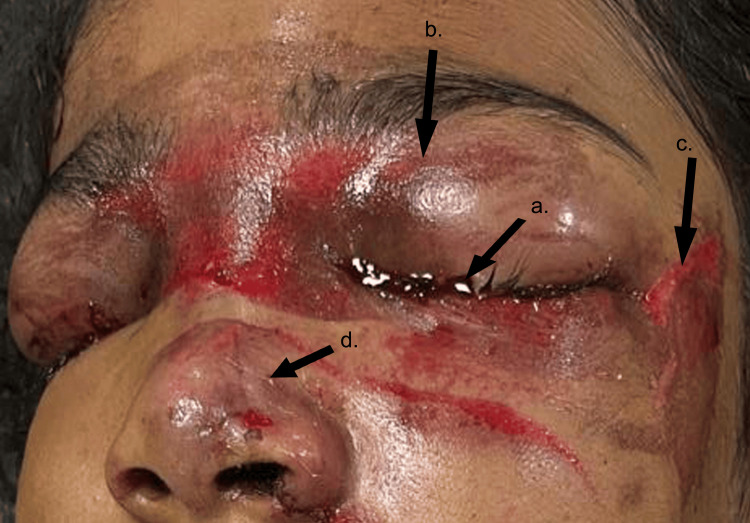
Facial injuries in case 2 (a) Full-thickness lid laceration. (b) Peri-orbital edema. (c) Superficial burn with redness and signs of inflammation. (d) Multiple abrasions. Informed consent was obtained from the patient’s legal guardian for the publication of the patient’s clinical information and images in this open-access journal.

A nine-year-old girl presented with stable vital signs, breathing spontaneously with oxygen saturation of 95% on room air, and a GCS score of 15. The E-FAST scan was negative. On external examination, first-degree burn marks were observed in the upper half of the left side of the face with redness, pain, tenderness, and mild swelling. There were multiple corresponding reddish scabbed abrasions over an area measuring 14.6 cm x 9.4 cm, situated just below the supraorbital ridges and above the level of the base of the nose. Lacerated wounds, measuring 1.8 cm x 1 cm and 1.5 cm x 0.8 cm, were observed on the left upper and lower eyelids, respectively, extending into the subcutaneous tissue. Fresh bleeding was observed at both nostrils. There was extensive swelling in both periorbital regions, accompanied by redness and signs of inflammation; however, no blisters were observed (Figure 2).

**Figure 3:**
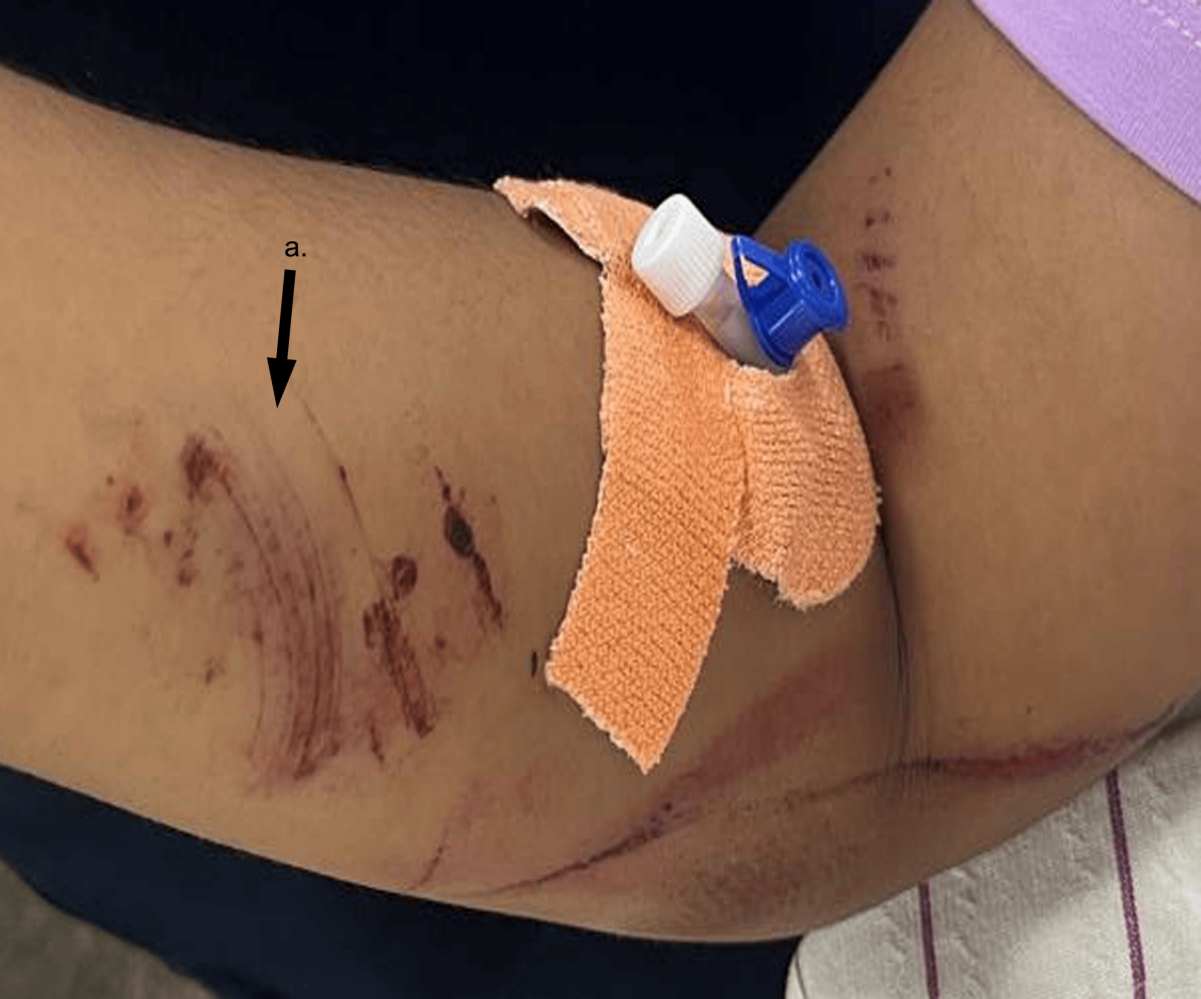
Multiple abrasions on the right forearm in case 2

Additionally, first-degree burn marks with redness and signs of inflammation were observed at the back of the lower one-third of the left forearm, extending to involve the back of the left hand, over an area of 6.8 cm x 5.5 cm, and on the back of the right hand, over an area of 3.1 cm x 2 cm, situated at the back of the proximal phalanx of the middle finger. Multiple reddish abrasions were observed on the proximal half of the front of the right forearm, over an area measuring 12.1 cm x 4.9 cm (Figure 3). All other systemic examinations were within normal limits. Non-contrast computed tomography (NCCT) of the face showed a fracture of the left ethmoid's lateral wall and both nasal bones, with no other skeletal or intracerebral injuries noted. After a detailed assessment, no active neurosurgical intervention was required. Ocular examination revealed periorbital edema, ecchymosis, and chemosis in both eyes. The left eye also showed traumatic mydriasis, grade 2 hyphema involving blood occupying approximately one-third of the anterior chamber, and a full-thickness upper lid laceration, which was repaired under local anesthesia. The NCCT orbital scan showed normal globe contour for both eyes. After evaluation and treatment, injectable antipyretics, topical antibiotics, and steroids were prescribed.

Both children were discharged after one day of observation and were advised to return to the outpatient department for follow-up. They were monitored closely, with formal clinical and ophthalmologic follow-up assessments conducted at one, three, and six months post-injury. At the final follow-up visit, all superficial burns and abrasions had healed completely in both children, and there was complete resolution of hyphema with minimal scar formation in the upper eyelid in case 2 without any effect on the child’s visual acuity. No long-term complications, such as secondary glaucoma, cataracts, or restricted ocular motility, were observed in either child.

## Discussion

Airbag deployment is vital for improving vehicle safety, but it can also cause a variety of injuries, especially in MVCs. Studies have shown that when children are seated in the front passenger seat and exposed to passenger-side airbag deployment, the overall risk of sustaining any injury rises to approximately 86%, compared to 55% for those unexposed. Such children experience a significantly higher rate of clinically significant serious injuries (14%) compared to unexposed cohorts (7.5%) [14]. These injuries may include burns, ocular trauma, and blunt cerebrovascular injuries [15]. Recognizing the types and consequences of these injuries is key to enhancing both safety protocols and medical treatment. For optimal safety and effectiveness, an airbag must inflate quickly enough to fully deploy before the occupant moves forward during a collision. Side-impact airbags inflate even more rapidly due to the limited space between the occupant and striking objects, such as another vehicle, a tree, or a pole [2]. Once deployed, the airbag deflates quickly, absorbing energy and cushioning the occupant to protect them from injury [16].

The airbag deployment process follows a sequence of events. During an accident, sudden deceleration or impact at about 20 km/h or more within a 60° frontal arc triggers crash sensors throughout the vehicle. These sensors send an electrical signal to the airbag cartridge, which contains sodium azide and an oxidizing agent [16]. The signal initiates the combustion of sodium azide, producing nitrogen gas and other byproducts, such as ammonia, carbon dioxide, and nitric oxide. These gases rapidly inflate the nylon rubber airbag within milliseconds. Afterward, the airbag deflates through vents, directing gases away from the occupant. Airbag capacities vary: the driver's side holds about 60 liters of air and the passenger side about 140 liters. The driver-side airbag expands to a depth of 25-30 cm, while the passenger-side airbag expands even further due to the greater available space. Tethered airbags are specialized airbag systems that use fabric straps or tethers sewn inside the airbag to control its shape, inflation speed, and deployment direction, thereby limiting its expansion. Untethered airbags, on the other hand, lack such protective features [17]. In our case, untethered airbags were installed in the vehicle; upon a sudden jerk, both airbags must have deployed uncontrollably, resulting in a burst on the passenger side. Understanding this mechanism is important as it illustrates the balance between rapid inflation for protection and the risks of forceful expansion.

Airbag deployment can cause a range of injuries, including ocular trauma, burns, and cervical spine injuries. Ocular injuries such as retinal detachment and corneal perforation have been reported, particularly in cases of severe facial trauma from seemingly minor accidents [18,19]. Deployment can also result in chemical and thermal burns, often affecting the upper extremities, with studies showing a higher incidence among females and most injuries being superficial partial thickness [20-25]. While airbags generally reduce cervical spine injuries, they may paradoxically increase the risk of blunt cerebrovascular injury in some cases. An airbag deployment event can also cause severe injuries when the occupants are not properly restrained, particularly in vulnerable populations. Children and smaller adults are at increased risk because they are more susceptible to the high forces exerted during deployment. A study reported that 159 out of 263 pediatric airbag-related injuries were fatal, with head trauma being the most common [26,27]. Cervical spine injuries, including cervical central spinal cord syndrome, have also been documented, especially in unrestrained individuals [28,29].

In this case, apart from the facial laceration caused by the blunt force impact of the airbag, the burn injuries on the back of the left forearm and the back of the right hand of the girl suggest that there was direct contact with the corrosive material of the airbag upon bursting. This occurred as she raised both her forearms in defense, placing them horizontally in front of her and her brother’s lower face. Since the boy's head was turned towards his father, who was seated in the driver’s seat, the right side of his face was completely spared. This mechanism of injury was postulated by the pattern of injuries, i.e., clear demarcation of facial injuries, observed only in the upper half of both children’s faces and later confirmed by the parents' statement upon inquiry. Abrasions observed on the inner aspect of the right forearm could have resulted from friction against the vehicle interior. The presence of fractures of the facial bones indicates that this injury was grievous in nature, as per section 116(g) of the Bhartiya Nyaya Sanhita (BNS) under Indian law [30].

Since out of six airbags, only two of them, located on the driver and front-passenger side, were deployed, that too in the absence of an impact or collision; this suggests the possibility of a non-collision system malfunction in the vehicle that may have triggered only these two sensors for deployment despite the low threshold. It was also informed that the vehicle had been sent for servicing three months ago and that no defect or malfunction was detected requiring rectification. However, the vehicle inspection report for the incident was not obtained, nor was an independent assessment by an automobile engineer or analysis by the authors conducted to confirm this. It cannot be denied that, in addition to being unrestrained, non-compliance with the specifications regarding the seating position of both children seems to be a major factor responsible for their injuries.

The risk of airbag deployment-related injuries can be reduced by following standard vehicle safety recommendations. Proper seatbelt use is essential, as airbags are designed to work in conjunction with them for maximum protection. Maintaining a safe seating position, at least 10 inches from the steering wheel or dashboard, helps minimize the risk of impact injuries [31]. Children under 12 should always ride in the back seat with appropriate restraints to avoid airbag-related harm. In specific cases, deactivating passenger airbags can protect small adults or children. Regular vehicle maintenance ensures that airbag sensors function correctly, while avoiding dashboard obstructions prevents interference with deployment. Safe driving practices further reduce unintended activation [32]. Public awareness and education on airbag safety are crucial for maximizing their protective benefits while minimizing potential harm. By adhering to these preventive measures, the effectiveness of airbags can be optimized, ensuring they serve their intended purpose of saving lives and reducing injury severity.

## Conclusions

Airbags play a crucial role in vehicle safety. At the same time, they are essential components intended to protect occupants in MVCs; they can also cause a variety of injuries during deployment, including burns, ocular trauma, cervical spine injuries, and blunt cerebrovascular injuries. The clinical findings in the two reported cases underscore the critical vulnerability of children when seated in the front row, particularly when improper restraint systems or standard vehicle safety recommendations are bypassed. These cases emphasize the clear anatomical and kinetic risks posed by the deployment forces of standard airbags to pediatric passengers. While the immediate and midterm management of these specific patients yielded stable ocular recovery, clinicians must remain vigilant regarding the acute presentation of the injury patterns. Ultimately, these cases reinforce the ongoing need for strict adherence to established child passenger safety guidelines, specifically rear-seat placement and appropriate booster seat utilization, to mitigate severe trauma risks.

## References

[1] (1993). The National Highway Traffic Safety Administration traffic safety plan for older persons. ROSA P.

[2] Nayak R, Padhye R, Sinnappoo K, Arnold L, Behera BK (2013). Airbags. Text Prog.

[3] Almahmoud T, Barss P (2014). Vehicle occupant restraint systems impact on eye injuries: a review. Surv Ophthalmol.

[4] Kenney KS, Fanciullo LM (2005). Automobile air bags: friend or foe? A case of air bag-associated ocular trauma and a related literature review. Optometry.

[5] Parvez MS, Rahman MM, Samykano M, Ali MY (2024). Current advances in fabric-based airbag material selection, design and challenges for adoption in futuristic automobile applications. Mater Today Proc.

[6] A guide to diagnosis and management. Diseases and Injuries to the Head, Face and Neck.

[7] Smith GC (2021). Reconstructing vehicle and occupant motion from EDR data in high yaw velocity crashes. SAE WCX Digital Summit.

[8] U.S. Department of Transportation, Federal Transit Administration (1-279). Research on vehicle crash: sensors and deployment thresholds in non-collision structural impacts. Transit Noise and Vibration Impact Assessment Manual.

[9] (2026). New inserts address rear leaf spring sounds. https://gm-techlink.com/?p=15234.

[10] Kumar A, Shrivastava A (2012). Safety-critical failure analysis of industrial automotive airbag system. Int J Adv Res Comput Sci.

[11] Corro LM, Williams AJ, Kimble RM, Griffin BR (2022). Burns in pediatric trauma patients. Clin Surg.

[12] Marshall KW, Koch BL, Egelhoff JC (1998). Air bag-related deaths and serious injuries in children: injury patterns and imaging findings. AJNR Am J Neuroradiol.

[13] Bhatti DS, Khan MA, Rodriguez DU, Cadogan J, Burge T (2021). Paediatric burns from deployment of a concealed aviation seatbelt airbag. Cureus.

[14] Durbin DR, Kallan M, Elliott M, Arbogast KB, Cornejo RA, Winston FK (2002). Risk of injury to restrained children from passenger airbags. Annu Proc Assoc Adv Automot Med.

[15] Stewart TC, Girotti MJ, Nikore V, Williamson J (2003). Effect of airbag deployment on head injuries in severe passenger motor vehicle crashes in Ontario, Canada. J Trauma.

[16] Ruff C, Jost T, Eichberger A (2007). Simulation of an airbag deployment in out-of-position situations. Veh Syst Dyn.

[17] Reed MP, Jermakian MJ, Neale AS (2009). Validation methodology on airbag deployment process of driver side airbag. 21st International Technical Conference on the Enhanced Safety of Vehicles (ESV).

[18] Duma SM, Jernigan MV (2003). The effects of airbags on orbital fracture patterns in frontal automobile crashes. Ophthalmic Plast Reconstr Surg.

[19] Koisaari T, Leivo T, Sahraravand A, Haavisto AK, Sulander P, Tervo TM (2017). Airbag deployment-related eye injuries. Traffic Inj Prev.

[20] Brumbelow ML, Jermakian JS (2022). Injury risks and crashworthiness benefits for females and males: which differences are physiological?. Traffic Inj Prev.

[21] Erpenbeck SP, Roy E, Ziembicki JA, Egro FM (2021). A systematic review on airbag-induced burns. J Burn Care Res.

[22] Jernigan MV, Rath AL, Duma SM (2004). Analysis of burn injuries in frontal automobile crashes. J Burn Care Rehabil.

[23] Skibba KE, Cleveland CN, Bell DE (2021). Airbag Burns: An unfortunate consequence of motor vehicle safety. J Burn Care Res.

[24] Monkhouse SJ, Kelly MD (2008). Airbag-related chest wall burn as a marker of underlying injury: a case report. J Med Case Rep.

[25] Cunningham K, Brown TD, Gradwell E, Nee PA (2000). Airbag associated fatal head injury: case report and review of the literature on airbag injuries. J Accid Emerg Med.

[26] Quiñones-Hinojosa A, Jun P, Manley GT, Knudson MM, Gupta N (2005). Airbag deployment and improperly restrained children: a lethal combination. J Trauma.

[27] Huber CD, Lee JB, Yang KH, King AI (2005). Head injuries in airbag-equipped motor vehicles with special emphasis on AIS 1 and 2 facial and loss of consciousness injuries. Traffic Inj Prev.

[28] El-Menyar A, Consunji R, Asim M (2016). Underutilization of occupant restraint systems in motor vehicle injury crashes: a quantitative analysis from Qatar. Traffic Inj Prev.

[29] Pearlman JA, Eong KA, Kuhn F, Pieramici DJ (2001). Airbags and eye injuries: epidemiology, spectrum of injury, and analysis of risk factors. Surv Ophthalmol.

[30] Government of India, Ministry of Home Affairs (2023). The Bharatiya Nyaya Sanhita, 2023 (Official Draft/Act Text).

[31] Deng YC (1995). Airbag burns: an unfortunate consequence of motor vehicle safety. 39th Stapp Car Crash Conference.

[32] Shaikh TN, Chaudhari S, Rasania H (2013). Air bag: a safety restraint system of an automobile. Int J Eng Res Appl.

